# The validity of the canadian triage and acuity scale in predicting resource utilization and the need for immediate life-saving interventions in elderly emergency department patients

**DOI:** 10.1186/1757-7241-19-68

**Published:** 2011-11-03

**Authors:** Ju Young Lee, Sang Hoon Oh, Eun Hee Peck, Jung Min Lee, Kyu Nam Park, Soo Hyun Kim, Chun Song Youn

**Affiliations:** 1 Emergency Team, Emergency Medical Center, Seoul St. Mary's Hospital, College of Medicine, The Catholic University of Korea, Seoul, Korea; 2 Department of Emergency Medicine, College of Medicine, The Catholic University of Korea, Seoul, Korea

**Keywords:** triage, geriatrics, emergency treatment

## Abstract

**Background:**

We evaluated the validity of the Canadian Triage and Acuity Scale (CTAS) in elderly emergency department (ED) patients. In particular, we examined the sensitivity and specificity of the CTAS for identifying elderly patients who received an immediate life-saving intervention in the ED.

**Methods:**

We reviewed the medical records of consecutive patients who were 65 years of age or older and presented to a single academic ED within a three-month period. The CTAS triage scores were compared to actual patient course, including disposition, discharge outcome and resource utilization. We calculated the sensitivity and specificity of the CTAS triage for identifying patients who received an immediate intervention.

**Results:**

Of the 1903 consecutive patients who were ≥ 65 years of age, 113 (5.9%) had a CTAS level of 1, 174 (9.1%) had a CTAS level of 2, 1154 (60.6%) had a CTAS level of 3, 347 (18.2%) had a CTAS level of 4, and 115 (6.0%) had a CTAS level of 5. As a patient's triage score increased, the severity (such as mortality and intensive care unit admission) and resource utilization increased significantly. Ninety-four of the patients received a life-saving intervention within an hour following their arrival to the ED. The CTAS scores for these patients were 1, 2 and 3 for 46, 46 and 2 patients, respectively. The sensitivity and specificity of a CTAS score of ≤ 2 for identifying patients for receiving an immediate intervention were 97.9% and 89.2%, respectively.

**Conclusions:**

The CTAS is a triage tool with high validity for elderly patients, and it is an especially useful tool for categorizing severity and for recognizing elderly patients who require immediate life-saving intervention.

## Background

Emergency department (ED) overcrowding is a serious problem worldwide [1-4]. The increase in the length of waiting lists for hospital admission in ED together with an increasing influx of new patients can cause severe overcrowding [5]. Meanwhile, in several countries (including South Korea), the exacerbation of an ageing society due to an increase in life expectancy is on the rise and is a social problem; in addition, ED visits by the elderly are increasing as well. What is characteristic among elderly patients is that their conditions are often more severe and they have a higher rate of admission to hospital compared to other age groups [6,7]. Often physiological signs or symptoms that are caused by the disease are hidden in initial triage. Accordingly, even in overcrowding situations, it is important to use a reliable triage system to determine which elderly patients who visit the ED require prompt emergency care and to provide the appropriate emergency treatment.

Among the various emergency patient triage systems, five-level triage systems such as the Emergency Severity Index (ESI) and the Canadian Triage and Acuity Scale (CTAS) are more widely used than three- or four-level triage systems. The CTAS is recognized as an accurate and reliable tool for rapid patient assessment [8]. Its reliability and validity has been demonstrated in both children and adults. It is extremely important to predict the need for immediate life-saving treatment among elderly emergency patients, who have more diverse presentations than other age groups; however, to date, no study has evaluated the use of the CTAS, which provides more information regarding early treatment than the ESI (which is a triage tool that predicts ED resource allocation). Therefore, we examined the validity of the CTAS as used by triage nurses on elderly patients who visited our ED during the research period. In addition, we assessed the sensitivity and specificity of the CTAS as a triage tool that can make predictions for elderly emergency patients who need immediate life-saving intervention.

## Methods

### Study design

The accuracy of the CTAS levels that were recorded by the triage nurse at the time of the initial patient visit was examined through a retrospective review of electronic medical records of patient treatment. The medical records included ED disposition, hospital discharge outcome and resource utilization, as well as whether immediate life-saving treatment was evaluated. This study was approved by the Institutional Review Board of the Catholic University of Korea, Seoul Saint Mary's Hospital.

### Setting and selection of participants

This study was conducted at an ED of a tertiary university hospital in Seoul that is visited by more than 60,000 patients annually, has a high number of chronically ill patients and is very chaotic. The study subjects were patients 65 years or older who had visited the ED from July, 2009 to September, 2009. We excluded patients who passed away before arrival, patients who visited for the issuance of a medical certificate (or other non-treatment purposes) and patients who discharged themselves against medical advice.

### Study protocol

The ED that conducted the study performed triage has used the CTAS since May 2008 for all patients who were admitted for treatment, and beginning in April 2009, the revised CTAS was used as the triage method (Table 1). All of the triage nurses began performing triage after at least two years of work experience at an ED; in addition, prior to performing their triage duties, they received theoretical training regarding the concepts, objectives and methods of triage and the role and qualifications of triage nurses and CTAS standards. After verification by an emergency medicine specialist using 20 randomly extracted cases from actual emergency room patients, the nurses began their triage duties and accessed to paper support when assigning a CTAS score. During the study period, a total of 20 nurses performed triage.

**Table 1:** Canadian Emergency Department Triage and Acuity Scale (CTAS) level

1.	Resuscitation
	• Conditions that threats to life or limb (or imminent risk of deterioration) requiring Immediate aggressive interventions.
	• Time to physician immediate
2.	Emergent
	• Conditions that are a potential threat to life, limb or function, requiring rapid medical intervention or delegated acts.
	• Time to physician assessment/interview ≤ 15 min
3.	Urgent
	• Conditions that could potentially progress to a serious problem requiring emergency intervention. May be associated with significant discomfort or affecting ability to function at work or activities of daily living
	• Time to physician ≤ 30 min
4.	Less urgent/Semi-urgent
	• Conditions that related to patient age, distress, or potential for deterioration or complications would benefit from intervention or reassurance within 1-2 hours
	• Time to physician ≤ 1 hours
5.	Nonurgent
	• Conditions that may be acute but non-urgent as well as conditions which may be part of a chronic problem with or without evidence of deterioration. The investigation or interventions for some of these illnesses or injuries could be delayed or even referred to other areas of the hospital or health care system.
	• Time to physician ≤ 2 hours

For verification of the validity of the CTAS scores, two researchers who were not familiar with the triage records of the subject patients independently reviewed the ED medical records to examine whether the patients were discharged or admitted to the intensive care unit (ICU) or hospital ward after the ED treatment or died in the ED and to determine whether immediate life-saving intervention was performed. In addition, all medical records--including the admission records--were independently reviewed to examine whether the patients died prior to hospital discharge, as well as the length of stay (LOS), hospital cost, specialist consultations and use of computed tomography (CT) scans from the ED visit to the time of discharge. When disagreement arose during the medical record review process, another researcher was consulted to make the decision and independently adjudicated for the disagreements. If the patient was transferred to another hospital due to circumstances at the hospital ward or the patient's wish to move even though admission to hospital after the ED treatment was necessary, then this was classified as an "admission"; however, if the patient transferred to another hospital due simply to the patient wishes, this was classified as a "discharge". Life-saving interventions are defined as interventions and treatments that were performed on a patient whose life was considered difficult to preserve or who would suffer serious mental and physical harm without the necessary emergency treatment due to illness, childbirth, damage caused by various accidents or disasters and other emergency situations; we used the standards that are presented in the ESI: Implementation Handbook (Ver. 4) [9]. (see Table 2). The use of immediate life-saving intervention is defined as a situation in which treatment was performed within an hour of the patient's arrival [10].

**Table 2:** Study definition of an immediate life-saving intervention

1.	Airway and breathing support, including intubation or emergent noninvasive positive pressure ventilation.
2.	Electrical therapy, including defibrillation, emergent cardioversion, or external pacing.
3.	Procedures, including chest needle decompression, pericardiocentesis, or open thoracotomy.
4.	Hemodynamic support, including significant intravenous fluid resuscitation in the setting of hypotension, blood administration, or control of major bleeding.
5.	Emergency medications, including naloxone, dextrose, atropine, adenosine, epinephrine, or vasopressors

Nurses with ten years of experience performed triage after reviewing the medical records to verify the degree of agreement among the triage nurses regarding 20 randomly extracted cases from among the elderly patients who were admitted during the study period.

### Data analysis

The distribution of patient baseline characteristics is presented as either a percentage or the mean ± SD. To compare the distribution of the baseline characteristics among triage scores, we used the ANOVA test for continuous variables, the Chi-square test or the Fisher's exact test for categorical variables. Non-normally distributed continuous variables were compared according to median values and tested for statistical significance using the Mann-Whitney test. Finally, correlation between each categorical outcome and CTAS score was evaluated by multivariate logistic regression analysis and odds ratios (OR) and 95% confidence intervals (CI) were compared with this CTAS level. The sensitivity, specificity, Positive predictive value (PPV) and negative predictive value (NPV) of the CTAS score for identifying patients who received an immediate intervention were calculated. The inter-rater reliability between triage nurses and the expert triage nurse was determined using the weighted kappa statistic. All statistical analyses were performed using SPSS 16 (SPSS, Chicago, IL), and differences with a *P*-value of < 0.05 were considered to be statistically significant.

## Results

### Patient characteristics and triage scores

**Figure 1:**
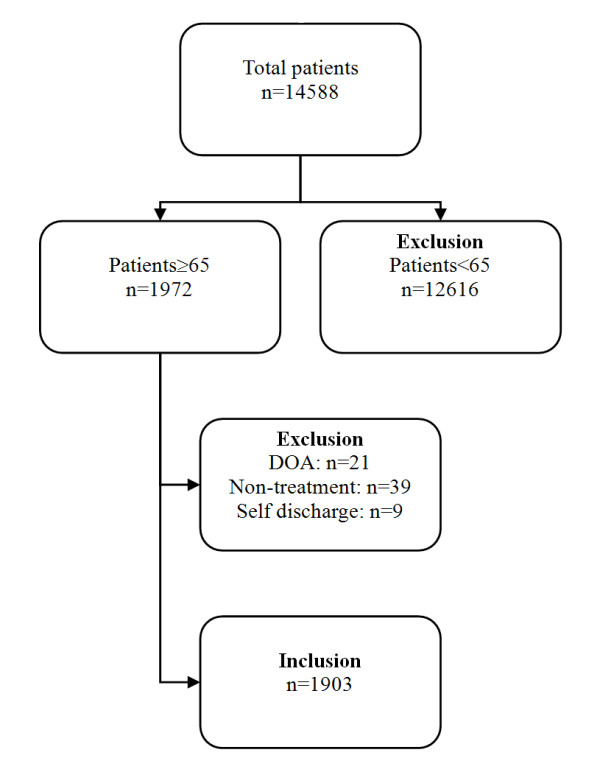
**Template for transferred cardiac arrest patients from other facility**. DOA: Death on arrival.

**Figure 2:**
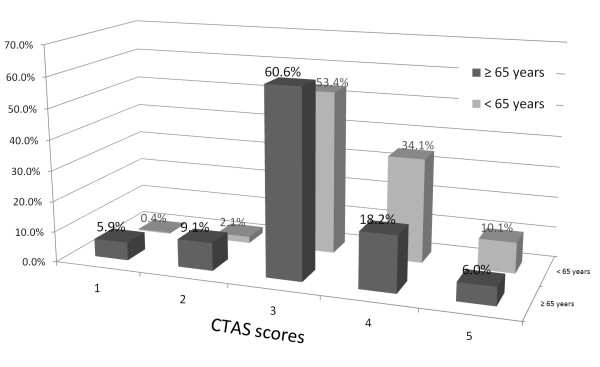
**Distribution of Canadian Triage and Acuity Scale (CTAS) score in patients who were ≥ 65 years of age and patients who were < 65 years of age**.

Among the 14,588 patients who visited the ED, 1972 patients were 65 years or older; after excluding 69 of these patients (21 patients passed away before arrival, 39 patients visited for non-treatment purpose, 9 patients discharged themselves), a total of 1903 patients were included in the study (Figure 1). The characteristics of the 1903 patients are shown in Table 3. 36.4% of the subjects were admitted to the hospital ward after ED treatment, and 10.2% of these patients were admitted to the ICU, which is significantly different than patients under the age of 65 (*P *< 0.001). The triage results for the subjects included 113 patients (5.9%) in level 1, 174 patients (9.1%) in level 2, 1154 patients (60.6%) in level 3, 347 patients (18.2%) in level 4 and 115 patients (6.0%) in level 5. In contrast, the patients under 65 years of age had triage levels 1 through 5 with 49 patients (0.4%), 245 patients (2.1%), 6353 patients (53.4%), 4055 patients (34.1%) and 1205 patients (10.1%), respectively; the ratio of level 1, 2 and 3 patients was significantly higher in the elderly patients (Figure 2). The degree of agreement between the expert nurses and triage nurses for the triage scales of 20 randomly extracted elderly patients from the subject patients was 86.2% (Kappa = 0.69 [95% CI = 0.68 to 0.71]).

**Table 3:** Demographic characteristics, dispositions and Canadian Triage and Acuity Scale (CTAS) triage levels for all patients

Characteristics	Patients ≥ 65 yr (n = 1903)	Patients < 65 yr (n = 11907)	*p*
Gender			
Female	1032 (54.2)	5637 (47.3)	< 0.001
Male	871 (45.8)	6270 (52.7)	
Age (Mean ± SD)	74.3 ± 7.0	29.9 ± 19.0	< 0.001
Disposition			
Discharged	1010 (53.1)	9667 (81.2)	< 0.001
Admitted-non ICU	692 (36.4)	2009 (16.9)	
Admitted-ICU	194 (10.2)	224 (1.9)	
Death	7 (0.4)	7 (0.1)	
CTAS Level			
1	113 (5.9)	49 (0.4)	< 0.001
2	174 (9.1)	245 (2.1)	
3	1154 (60.6)	6353 (53.4)	
4	347 (18.2)	4055 (34.1)	
5	115 (6.0)	1205 (10.1)	

Parenthesis indicates percentage.

ICU: intensive care unit

### Severity among triage scores (disposition and discharge outcome) (Table 4)

**Table 4:** Disposition, discharge outcome and resource utilization of each Canadian Triage and Acuity Scale (CTAS) for elderly patients

			CTAS			*p*
	
	Level 1 (n = 113)	Level 2 (n = 174)	Level 3 (n = 1154)	Level 4 (n = 347)	Level 5 (n = 115)	
Disposition						
Discharge	3 (2.7)	32 (18.4)	584 (50.6)	281 (81.0)	110 (95.7)	< 0.001
Admission-non ICU	20 (17.7)	70 (40.2)	531 (46.0)	66 (19.0)	5 (4.3)	
Admission-ICU	85 (75.2)	70 (40.2)	39 (3.4)	0 (0.0)	0 (0)	
Death	5 (4.4)	2 (1.1)	0 (0.0)	0 (0.0)	0 (0)	
Discharge outcome						
Alive	89 (78.8)	142 (81.6)	1121 (97.1)	347 (100.0)	115 (100.0)	< 0.001
Death	24 (21.2)	32 (18.4)	33 (2.9)	0 (0.0)	0 (0.0)	
LOS (day)*	9.0 (4, 20)	5.0 (1, 12)	1.3 (0, 8)	0 (0, 1)	0 (0, 0)	< 0.001
Consult	84 (74.3)	102 (58.6)	480 (41.6)	58 (16.7)	4 (3.5)	< 0.001
CT	72 (63.7)	98 (56.3)	485 (42.0)	97 (28.0)	11 (9.6)	< 0.001

*Median (Interquartile rage)

Parenthesis indicates percentage.

ICU: intensive care unit

LOS: length of stay

CT: computed tomography

**Figure 3:**
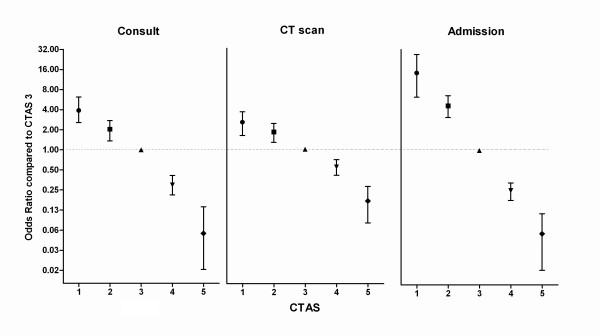
**Odds ratio for consultation, CT scan and admission by Canadian Triage and Acuity Scale (CTAS) score**. Each plot represents the odds ratio and 95% confidence interval compared with CTAS 3.

Among the elderly patients who were classified as CTAS level 1, the admission rate was 92.9% and the ICU admission rate was 75.2%; five of these patients (4.4%) died in the ED, and twenty-four (21.2%) died prior to hospital discharge. Among the elderly patients who were classified as CTAS level 2, the admission rate was 80.5%, and the ICU admission rate was 40.2%; two of these patients (1.1%) died in the ED, and thirty-two (18.4%) died prior to hospital discharge. Among the elderly patients who were classified as CTAS level 3, the admission rate was 49.4%, and the ICU admission rate was 3.4%; none of these patients died in the ED, and thirty-three (2.9%) died prior to hospital discharge. The admission rates for level 4 and level 5 patients were 19.0% and 4.3%, respectively, and no patients in either group were admitted to the ICU and died in the ED or prior to hospital discharge. Compared with patients in CTAS 3, the OR for an admission was 12.99 (95% CI = 6.26 to 26.95), 4.15 (95% CI = 2.80 to 6.16), 0.25 (95% CI = 0.18 to 0.33), 0.05 (95% CI = 0.02 to 0.11) for patients in CTAS 1, 2, 4, and 5, respectively (Figure 3).

### Resource utilization among triage scores

**Figure 4:**
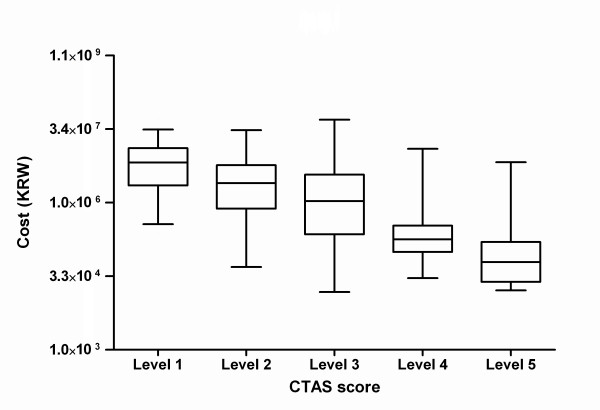
**Box and whisker plot of hospital cost by Canadian Triage and Acuity Scale (CTAS) score**. Bars represent median, boxes represent the interquartile range, and whiskers extend to 5th and 95th percentiles.

There was a significant correlation between CTAS score and the odds of specialist consultation and CT scan (Figure 3). Compared with patients in CTAS 3, the ORs for consultation were 3.98 (95% CI = 2.56 to 6.19), 1.97 (95% CI = 1.42 to 2.73), 0.29 (95% CI = 0.21 to 0.39), 0.05 (95% CI = 0.02 to 0.14) for patients in CTAS 1, 2, 4, and 5, respectively. Compared with patients in CTAS 3, the OR for a CT was 2.41 (95% CI = 1.61 to 3.61), 1.76 (95% CI = 1.27 to 2.43), 0.54 (95% CI = 0.41 to 0.70), 0.15 (95% CI = 0.08 to 0.28) for patients in CTAS 1, 2, 4, and 5, respectively. There was a significant difference between the CTAS score and hospital cost when analyzed using an ANOVA (*P *< 0.001) (Figure 4).

### Immediate life-saving intervention in elderly patients (Table 5)

**Table 5:** Immediate life-saving intervention versus demographic characteristics, dispositions and CTAS Triage level for elderly patients

	Immediate intervention		
		
Characteristics	Received (n = 94)	Non received (n = 1809)	*p*
Gender			
Female	52 (55.3)	980 (54.2)	0.828
Male	42 (44.7)	829 (45.8)	
Age			
Age 65-74, yr	39 (41.5)	1072 (59.3)	< 0.001
Age 75-84, yr	27 (28.7)	569 (31.5)	
Age ≥ 85, yr	28 (29.8)	168 (9.3)	
Disposition			
Discharged	8 (8.5)	1002 (55.4)	< 0.001
Admitted-non ICU	27 (28.7)	665 (36.8)	
Admitted-ICU	53 (56.4)	141 (7.8)	
Death	6 (6.4)	1 (0.1)	
Discharge outcome			
Alive	59 (62.8)	1755 (97.0)	< 0.001
Death	35 (37.2)	54 (3.0)	
CTAS Level			
1	46 (48.9)	67 (3.7)	< 0.001
2	46 (48.9)	128 (7.1)	
3	2 (2.1)	1152 (63.7)	
4	0 (0.0)	347 (19.2)	
5	0 (0.0)	115 (6.4)	

Parenthesis indicates percentage.

ICU: intensive care unit

A total of 94 patients received immediate life-saving intervention. Among these 94 patients, 42 (44.7%) were male and 52 (55.3%) were female. The average ages for male and female patients were 75.5 ± 8.5 and 79.6 ± 8.4 years, respectively. As the result of ED treatment, 53 patients (56.4%) were admitted to the ICU, twenty-seven patients (28.7%) were admitted to the hospital ward, eight patients (8.5%) were discharged from the hospital, and six patients (6.4%) died in the ED; 35 of the 94 patients (37.2%) died prior to hospital discharge. The triage scales for these 94 patients were 46 patients (48.9%) in level 1, 46 patients (48.9%) in level 2 and 2 patients (2.1%) in level 3. Sensitivity, specificity, PPV and NPV were 48.9% (95% CI = 38.5% to 59.5%), 96.3% (95% CI = 95.3% to 97.1%), 40.7% and 97.3%, respectively, for immediate life-saving intervention for CTAS level 1, whereas sensitivity, specificity, PPV and NPV were 97.9% (95% CI = 92.5% to 99.7%), 89.2% (95% CI = 87.7% to 90.6%) and 32.1%, 99.9%, respectively, for a CTAS level ≤ 2. A total of 129 immediate life-saving interventions were performed on these 94 patients and twenty-six patients received at least two types of intervention. The most common interventions were hemodynamic support (68 patients), followed by intubation or emergent non-invasive positive pressure ventilation (41 patients), emergency medication (12 patients) and electrical therapy (eight patients). Of the 94 patients who received immediate life-saving intervention, 46 were classified as CTAS level 2, and this represents 26.4% of all CTAS level 2 patients in the study. The life-saving interventions that were performed on these 46 patients within one hour of their visit included hemodynamic support (38 patients), intubation or emergency non-invasive positive pressure ventilation (15 patients), electrical therapy (two patients) and/or emergency medication (two patients).

## Discussion

ED overcrowding leads to increases in waiting time, delays in treatment and a decline in the quality of emergency treatment. Furthermore, overcrowding increases the difficultly in promptly recognizing patients with severe conditions and compromises the ability to subsequently provide the proper treatment [5,11-14]. Elderly patients typically have a complicated underlying disease, and their disease may progress rapidly due to reduced immunity or a lack of self-awareness with regard to their condition [15]. In addition, these patients often cannot clearly explain their clinical condition or history, and the physiological signs or symptoms that are caused by the disease can also be hidden; therefore, obtaining information from these patients at the triage level can be difficult [16,17]. Several studies regarding geriatric trauma stressed that the mortality rate of elderly patients is approximately 2-6 times higher than in young and middle-aged adult patients because of the elderly characteristics; consequently, these studies emphasized the importance of prompt treatment and the significance of an accurate initial assessment and triage classification [18,19].

At present, five-level triage systems such as the ESI and the CTAS are widely used as emergency patient triage tools for accurately classification. Unlike other classification tools, the ESI is a comprehensive algorithm-type triage tool that not only includes acuity through symptoms and physiological indicators but also includes the use of expected resources, and there have been various studies regarding its validity and reliability in elderly patients. One study reported a strong correlation between triage results and the duration of stay in the ED, the number of hospitalization days and the use of resources [20]. However, another study reported that the ESI is not useful for predicting which elderly emergency patients will need immediate life-saving treatment, yielding a sensitivity and specificity of 42.3% and 99.2%, respectively [10]. Another five-level triage tool is the CTAS, which was first developed in 1990s. After a continuous revision process, the CTAS is currently used in Canada and many other countries. The CTAS has been recognized as a tool that can rapidly assess patients and is sensitive, accurate and reliable [21,22]. The validity and reliability of the CTAS as a triage system for both children and adults have been verified [23-27]. However, there are no studies to date regarding the validity of CTAS triage for elderly patients, in particular as a predictor of the necessity for immediate life-saving treatment.

In this study, the triage levels of 1903 elderly patients aged 65 years and older were 113 patients (5.9%) in level 1 and 174 patients (9.1%) in level 2; thus, the proportions of levels 1 and 2 among elderly patients were approximately five times higher than for patients under 65 years of age. In addition, the admission rate was 46.6% and 10.2% were admitted to the ICU, confirming that the severity and hospitalization rate for elderly patients were higher than for younger age groups, and this is consistent with the results of previous studies [10,20]. This finding is likely due to the increase in average life expectancy that has led to an increase in the elderly population and a subsequent increase in both chronic illnesses and their severity.

This study is the first study of the validity of triage classification of elderly emergency patient using the CTAS. In this study, there was a significant correlation between the CTAS score and the patient severity and resource utilization, and the patients who required and received a life-saving intervention within an hour following their arrival to the ED were almost classified as level 1 or level 2 patients. On the other hand, a previous study using the ESI reported that 23.1% of patients received immediate life-saving intervention was ESI level 3 [10]. This indicates that the ESI can potentially under-triage seriously ill elderly patients compared with the CTAS. Patients who were classified as CTAS level 2 but needed immediate life-saving intervention included patients who initially had stable vital signs but experienced a sudden change in their condition that necessitated large volume resuscitation due to hypotension and interventions for respiratory distress. The finding that many elderly patients can show a change in their condition within an hour of their visit to the hospital means there is a need to recognize not only patient's physical condition and vital signs at the time of the hospital visit but also possibility of condition change. If a life-saving intervention is suddenly performed on a patient in a low triage level, it is more likely to result in a poor outcome (e.g., the death of the patient). Of the 174 patients who were classified as level 2, 46 (26.4%) needed a life-saving intervention due to a change from their initial assessment; moreover, taking into account that these patients were elderly and had some characteristics that differed from other age groups, the patients in this category required frequent reassessment and careful monitoring for sudden changes in their hemodynamic status and blood oxygen saturation.

This study has a few limitations that bear mentioning. First, because this was a study using retrospective chart data, any patients who needed but did not receive immediate life-saving treatment were omitted. Second, although there was an effort to maintain a consistent quality of triage, triage was performed by many nurses and we did not determine CTAS reliability using 'real time' observational or blinded independent nurse triage. Third, this study was limited to an ED of a tertiary university hospital in Seoul, and the results may vary depending on the location and/or size of the hospital and its characteristics.

## Conclusions

This study demonstrates a strong correlation among CTAS scores, the severity of elderly patients (e.g., death and admission rate) and resource utilization (e.g., LOS, cost, consultation and the use of a CT scan). The correlation between CTAS Level ≤ 2 and the use of immediate life-saving intervention among elderly patients over 65 years of age was 97.9% sensitive and 89.2% specificity, with high predictability for immediate life-saving intervention.

## Abbreviations

CT: computed tomography; CTAS: Canadian Triage and Acuity Scale; ED -emergency department; ESI: Emergency Severity Index; ICU: intensive care unit; LOS: length of stay.

## Competing interests

The authors declare that they have no competing interests.

## Authors' contributions

JYL drafted the manuscript. EHP, KNP and SHK reviewed data and revised the manuscript. JML managed the data and reviewed critical revisions to the manuscript. SHO performed data analysis and revised the manuscript. CSY conceived the research and drafted the manuscript. All authors have read and approved the final manuscript.
